# How can we mainstream mental health in research engaging the range of Sustainable Development Goals? A theory of change

**DOI:** 10.1371/journal.pgph.0000837

**Published:** 2022-08-31

**Authors:** Anna Madill, Poornima Bhola, Erminia Colucci, Karina Croucher, Adrian Evans, Rebecca Graber

**Affiliations:** 1 School of Psychology, Lifton Terrace, University of Leeds, Leeds, United Kingdom; 2 Department of Clinical Psychology, National Institute of Mental Health and Neurosciences, Bangalore, India; 3 Department of Psychology, School of Science and Technology, Middlesex University London, Hendon, United Kingdom; 4 School of Archaeological and Forensic Sciences, University of Bradford, Bradford, United Kingdom; 5 School of Humanities and Social Science, University of Brighton, Brighton, United Kingdom; University of Washington, UNITED STATES

## Abstract

Mental health is a leading cause of ill-health worldwide, disproportionately affects low-and-middle-income countries and, increasingly, is considered relevant across the Sustainable Development Goals (SDGs). Hence, we ask: How can we mainstream mental health in research engaging the range of SDGs? We use the UK Research and Innovation Global Challenges Research Fund (GCRF) as a case study. In a previous scoping review, we purposefully sampled non-mental health focused GCRF grants for diversity from 2015 until May-end 2020 (*N* = 36). In the present study, the principal investigator of each grant in this sample was invited to interview (11 accepting). Snowballing, our networks, and returning to the funding archive secured a further 15 interviews sampled for diversity (Final sample: 13 UK researchers and 13 of their overseas collaborators). A thematic analysis of this data organised key information into a trajectory from the challenges of incorporating mental health impact, to how these challenges might be overcome and, finally, to support needs. This analysis was then organised into a Theory of Change designed to promote the mainstreaming of mental health in global challenges research. We outline the implications for global challenges researchers, mental health practitioners, and global challenge research funders. One important implication is that we provide evidence to encourage funders to engage with the desire of researchers to contribute more broadly to the wellbeing of the communities with whom they work.

## Introduction

The World Health Organisation (WHO) conceptualises mental health as “a state of well-being in which the individual realizes his or her own abilities, can cope with the normal stresses of life, can work productively and fruitfully, and is able to make a contribution to his or her community” [1 p1]. In a review of the evidence, the *Lancet Commission on Global Mental Health and Sustainable Development* [2] reports that mental health is one of the leading causes of ill-health worldwide and disproportionately affects low- and middle-income countries (LMIC). Moreover, the mental health workforce gap globally, and particularly in LMIC, is huge [3].

Mental health aligns most directly with United Nations (UN) Sustainable Development Goal (SDG) 3: Good Health and Well-Being, specifically target 3.4 which aims by 2030 to reduce by one third premature mortality from non-communicable diseases through prevention and treatment and the promotion mental health and well-being [4]. Increasingly, mental health is considered of fundamental relevance across the global challenges represented by the SDGs [5] with strong arguments for “a dramatic reframing of mental health as not just a health issue, but as a crosscutting development issue” [6 p19]. This broad vision is reflected in the priorities outlined by the *Lancet Commission*: i.e., to extend the global mental health agenda to the general population; integrate the global response within other priorities and engage a wide range of stakeholders beyond health; target social and environmental causes; and engage in innovative use of non-specialists and digital technologies to deliver mental health interventions.

The recently updated WHO *Comprehensive Mental Health Action Plan 2013–2030* [1] concurs with this vision in that two of its six cross-cutting principles are: (i) a multi-sectoral approach, and (ii) action on social determinants. This acknowledges that “integrating mental health across wider sectors such as education, employment and social welfare helps address social determinants of mental health and ensures a comprehensive and holistic approach” [7 p12]. Shen, Eaton and Snowden [8] investigated mental health mainstreaming in 42 countries in terms of a shift towards providing mental health care from institutional to community settings. They concluded that “different countries have adapted deinstitutionalization in ways to meet idiosyncratic situations and population needs” [7 p313] and that more needs to be done with regard to management and implementation strategies. While the keyword for Shen, Eaton and Snowden [8] is ‘deinstitutionalisation’, for Gómez-Dantés and Frenk [9] it is ‘integration,’ and they provide a dimensional approach with regard to what is involved in mainstreaming mental health: “(i) incorporating mental disorders to the global and local agendas related to NCDs [non-communicable diseases]; ii) moving away both from the biological and sociological reductionisms around mental health prevalent in the past century; iii) addressing the whole range of conditions related to mental health; iv) migrating from the idea that mental diseases have to be treated in secluded clinical spaces; and v) expanding the use of a comprehensive approach in the treatment of these disorders, which includes medication, psychotherapy and other types of therapies” [9 p214].

The ideologies and practices of mainstreaming are complex and contentious and include, but go beyond, deinstitutionalisation and integration. Arguably, the concept of mainstreaming first gained traction in the field of education in the 1980s with regard to the benefits of schooling children with special needs in the general classroom [10]. In global development, the idea of mainstreaming emerged in the area of gender inclusivity in the 1990s [11]. At the turn of the century, mainstreaming was being considered with regard to global mental health when, in an editorial for the *Bulletin of the WHO*, Üstün argued that “it’s time to move mental health into the mainstream of health policy and practice” [12 p412]. Üstün’s editorial proved prescient and in 2010 the UN-WHO Policy Analysis─*Mental Health and Development*: *Integrating Mental Health into All Development Efforts including Millennium Development Goals*─committed to actions embedded in evidence that “(p)oor mental health is both a cause and a consequence of poverty, compromised education, gender inequality, ill-health, violence and other global challenges” [13 para 3; see also 14].

The current article builds on previous work in which we explored the potential to embed psychosocial wellbeing impact in global challenges research where the primary aims are not mental-health-related. In short, we transfer the concept of mental health mainstreaming to development-oriented research. We used the terminology ‘psychosocial wellbeing’ because it allowed us to identify “where diverse projects rub-up against mental health through defining broadly what this means; mental-health-related activity in disciplines that do not employ a medical perspective but spans interest at the level of the individual, group, community and region; and routes to impact aligned with SDGs in domains including economic, demographic, environmental, social, and cultural” [15]. Given the impact we believe is in reach, our approach to mainstreaming mental health forefronts transformative social change with its emphasis on long-term, sustainable, and community-owned benefits.

We used the UK Research and Innovation (UKRI) Global Challenges Research Fund (GCRF) as a case study. The GCRF is structured around six global strategic challenge portfolios aligned with the SDGs: Cities & Sustainable Infrastructure; Education; Food Systems; Global Health; Resilience to Environmental Shocks & Change; Security, Protracted Conflict, Refugee Crises & Forced Displacement. Thirty-six non-mental-health GCRF grants were purposefully sampled for diversity and coded for relevant information. Our findings indicated that 50–70% of non-mental-health GCRF projects already engage implicitly, but non-strategically, with psychosocial wellbeing impact. This suggests there are relatively achievable opportunities to impact psychosocial wellbeing across SDGs through routine, research project activities. Doing so will require a culture change in the predominantly public sector constituted by global challenges research.

Lewis and Connors conclude, with regard to the Australian public sector, “it may be that behaviour change rather than complete culture change is the only option in the case of change externally imposed by government” [16 p159]. More optimistically, Somerville [17] argues, from a study of the Canadian federal government, that a shift in sector culture can be achieved in as little as three years with the right drivers: i.e., (i) changes in leadership personnel; (ii) turnover of personnel; (iii) changes in human resources practices; (iv) communication; and, (v) enabling changes in structure and processes. With the closest resonances to the current study, Baker et al address the impact of climate change, population growth, and urbanisation on water management, arguing that the sector requires a change in culture “to create a reliable, sustainable and resilient water service” [18 p2]. Using a similar methodology to our own, Baker et al employed grounded theory to analyse interviews with managers at five British water and sewerage companies. Themes were then mapped onto the ‘Safe & SuRe’ framework which outlines interventions enabling a more resilient system: i.e., mitigation, adaptation, coping, and learning.

In this article we ask: How can we mainstream mental health in research engaging the range of SDGs? We address our research question in two stages. First, we undertake a thematic analysis of 13 interviews with UK researchers with non-mental-health focused GCRF funding and 13 of their overseas collaborators. Second, we organise this analysis into a Theory of Change (ToC): a methodology for planning, participation, and evaluation designed to promote the mainstreaming of mental health in global challenges research.

## Methodology

### Ethics statement

Approval for this research was awarded by the Ethics Committee of the School of Psychology, University of Leeds, UK: PSYC-26 21st April 2020. Interviewees were provided with an information sheet and conditions of consent sheet when approached for interview. Prior to interview their acceptance of the conditions of consent was audio-recorded before the interview commenced as approved by the Ethics Committee.

### Sampling and participants

GCRF grants were scoped using information on the UKRI gateway to research (GtR: https://gtr.ukri.org/) [15]. GtR is a searchable database allowing analysis of information on publicly-funded UK research. It includes grants funded by the following organisations, all of which are independent, non-departmental public bodies of the UK Government’s Department for Business, Energy and Industrial Strategy: Arts and Humanities Research Council (AHRC), Biotechnology and Biological Sciences Research Council (BBSRC), Economic and Social Research Council (ESRC), Engineering and Physical Sciences Research (EPSRC), Medical Research Council (MRC), Natural Environment Research Council (NERC), and Science and Technology Facilities Council (STFC).

GCRF grants were excluded if their primary research aim was mental health, i.e. which were classified on GtR as ‘Mental Health’ Research Topic or Health Category (*N* = 36). The remaining grants, awarded from the beginning of the GCRF programme towards the end of 2015 until the end of May 2020, were sampled for diversity across research council, GCRF strategic challenge portfolio, and world region: Africa, Americas, Southeast Asia, Europe, Eastern Mediterranean, and Western Pacific (https://www.who.int/about/who-we-are/regional-offices). Where sample diversity allowed, closed (total pool *N* = 484) rather than active (total pool *N* = 740) grants were selected for completeness of available GtR information. Differentiation of lead organisation and Research Category was also sought (final *N* = 36).

The principal investigator (PI) of each of the 36 grants was emailed an invitation to take part in an interview. All who expressed interest were interviewed. One reminder email was sent to PIs of projects that would increase the diversity of our interview sample, specifically male PIs, PIs in LMIC, and PIs of STFC grants. This secured 11 interviews with UK PIs (one jointly with the grant research assistant), one with a co-investigator based in the UK at the time of the grant, and one with a non-UK-based PI. We snowball sampled by asking each of these interviewees to introduce us to a non-UK-based collaborator. In this way, we secured a further five interviews and snowballed again to a sixth. We then used our own GCRF networks and returned to the GtR to close gaps in our sampling, securing a further four interviews and snowballing to a fifth. Our networks and snowballing provided us access to two non-UK-based collaborators who are experts in mental health and it was decided to undertake these interviews to add this perspective to the project. In total, we conducted 13 UK-based interviews and 13 non-UK-based interviews across 18 GCRF grants, two of the non-UK-based interviewees associated more tangentially with the GCRF programme as networked collaborators. Interviewing stopped when sampling for diversity across relevant variables was met (Tables 1 & 2). This was analysed as one data set of 26 interviews which makes this a moderately large sample for this kind of qualitative study [19]. Saturation occurred at around 12–14 interviews, and subsequent data added detail and examples but no new themes. The six GCRF strategic portfolios can be clustered into four themes at a slightly higher level of conceptualisation and, for economy, we will use these four themes from this point on: i.e., Education, Health, People & Societies, and Planetary Health.

**Table 1:** Scoped projects by GCRF portfolio, research council, status, region and interviewee.

GCRF Strategic Challenge Portfolio		Research Council	Active/ Closed	Global Region UK PI^a^ interviewee	Non-UK-based interviewee
Education	Education (N = 5)	AHRC	A	Americas	/
				Eastern Mediterranean	/
				SE Asia	/
		ESRC	C	Africa	/
				Americas^b^	Collaborator
		/	/		Collaborator
Health	Health (N = 15)	AHRC	C	Americas/Eastern Mediterranean	3^rd^ sector collaborator, Americas, Education
		BBSRC	*A* ^d^	*Western Pacific*	*Chinese national RA*^e^ *based in UK*
			C	Africa	/
				Africa	/
		EPSRC	A	Africa^f^	/
				Americas^f^	/
				Europe	/
		ESRC	*A*	*SE Asia*	*CoI*^h^ *Mental Health*
			C	Africa	/
		MRC	A	Africa^fg^	/
				Western Pacific	Clinical Trials Unit based in SE Asia
			C	Americas/Western Pacific (CoI)	/
				Africa	Collaborator
				SE Asia^f^	PI
		NERC	C	Africa	/
				Africa/Western Pacific	/
				SE Asia	CoI British national based in SE Asia
		/	/	*Global*	*Director*, *International* *Mental Health NGO*^i^
People & Societies	Cities & Sustainable Infrastructure (N = 6)	AHRC	C	SE Asia	/
		EPSRC	A	Global (PI & RA)	/
		ESRC	A	Africa	/
				Global	/
			C	Africa/Americas/SE Asia/Western Pacific	/
				Eastern Mediterranean/ Europe/SE Asia	/
	Security Protracted Conflict, Refugee Crises and Forced Displacement (N = 1)	AHRC	C	Americas	/
			A	*Global*	*CoI (Europe)*
Planetary Health	Food Systems (N = 6)	BBSRC	A	Africa	/
				SE Asia	/
			C	SE Asia/Western Pacific	/
		*EPSRC*	/	*Western Pacific*	*Collaborator*
				*Africa*	*Collaborator*
		STFC	C	Africa	/
				Americas	/
				Eastern Med/SE Asia/ Western Pacific	/
	Resilience to Environmental Shocks & Change (N = 3)	NERC	C	Africa	/
		STFC	C	Americas	/
				Global	/

Note a: Principal Investigator; Note b: Fellowship; Note c: Dotted line indicated snowball recruitment from interviewee in higher box to interviewee in lower box; Note d: Italics indicate interviewee additional to scoped projects; Note e: Research Assistant; Note f: Non-UK lead organisation; Note g: Intramural; Note h: Co-Investigator; Note i: Non-Governmental Organisation.

**Table 2:** Interviews by gender, GCRF strategic portfolio, research council, and world region.

	UK	Non-UK
**Gender**		
Male	3	4
Female	11^a^	9
**GCRF Strategic Portfolio Theme**		
Education	1	3
Health	7	7
People & Societies	3	1
*Cities*	1	0
*Security*	2	1
Planetary Health	2	2
*Food*	1	2
*Environment*	1	0
**Research Council**		
AHRC	3	2
BBSRC	1	1
EPSRC	1	2
ESRC	2	2
MRC	3	3
NERC	3	1
STFC	0	0
Not applicable	0	2
**World Region** ^**b**^		
Africa	5	2
Americas	4	3
Southeast Asia	2	3
Europe	1	1
Eastern Mediterranean	1	1
Western Pacific	4	3
Global	2	1

Note a: One interview had two female participants.

Note b: All relevant mentions in Table 1.

### Ethical statement

Approval for this research was awarded by the Ethics Committee of the School of Psychology, University of [*omitted for review*], PSYC-26 21^st^ April 2020. Interviewees were provided with an information sheet and conditions of consent sheet when approached for interview. Prior to interview their acceptance of the conditions of consent was audio-recorded before the interview commenced as approved by the Ethics Committee.

### Data collection

Interviews were conducted online by the first author between October 2020 and July 2021. A semi-structured format was used such that the interviewer covered pre-planned areas relevant to the research question, while using follow-ups to elicit further detail and facilitating the interviewee to lead the topical flow [20]. The following questions were always covered: Can you tell me about your involvement in GCRF/international development work?; To what extent is there potential to incorporate mental health impact into the kind of work you do?; What do you see as the main challenges of incorporating mental health impact into the kind of work you do?; How might these challenges be overcome?; What support would enable you to incorporate mental health impact in your work?; To what extent do you think there is an appetite to incorporate mental health impact into the kind of work you do? Each interview lasted around one hour.

### Analytical procedures

The iteration of thematic analysis [21] appropriate to the research question and material was: a rich description of (most of) the data as opposed to a detailed account of one particular aspect; inductive (‘bottom-up’) as opposed to theoretical (‘top-down’); and a semantic as opposed to latent approach, meaning that analysis focused on the interviewees’ own words in context rather than seeking to deconstruct or to (overly) interpret meaning [22]. Small amounts of ‘top down’ theorising and interpretation were incorporated as the analysis developed in order to close gaps and link themes. This analysis is compatible with a critical realist position which accepts the existence of ‘objects’ and real impacts of social structures while considering human understanding of the world to be contextual and mediated always by language and culture [23].

It was found that most interview content relevant to the research question could be condensed around three of the interview questions: What do you see as the main challenges of incorporating mental health impact into the kind of work you do?, How might these challenges be overcome?, and What support would enable you to incorporate mental health impact in your work? Content relevant to each of these three questions was selected from across the data set and analysed separately. Theme development and labelling was refined until the most parsimonious number of themes were generated relevant to each of the three questions, such that Q1 has eight themes, Q2 has seven themes, and Q3 has five themes. Finally, the themes were inspected and a trajectory through the three questions became apparent: that is, the themes identified as the main challenges of incorporating mental health impact could be matched with the themes indicating how these challenges might be overcome and, finally, to themes identifying support needs related to each posited solution.

The project co-investigators and one project mentee then met online in three groups of 3–5 people (*N* = 13) to discuss the analysis and to provide written feedback to the first author. All co-investigators have experience of GCRF research, in many different world regions, and span multiple disciplines and methods. Feedback was also obtained from the project steering group consisting of a representative from each of our three Partner Organisations in LMIC with expertise in mental health. After the analysis had been refined, it was again presented at a project team meeting of 14 people and agreed as a useful, orienting overview addressing the research question.

## Results

Table 3 outlines the results, listing the themes identified for each of the three guiding questions organised across the table showing a trajectory from the challenges of incorporating mental health impact, to how these challenges might be overcome and, finally, to support needs.

**Table 3:** Overview of the results.

What do you see as the main challenges of incorporating mental health impact into the kind of work you do?	How might these challenges be overcome?	What support would enable you to incorporate mental health impact in your work?
Low priority	Leadership & stipulation	Advocacy
	Link to a ‘sellable’ priority &/or normal practices	
Evidence	Flexible methods	Data & evidence
Tools		Research tools
Timeframe	Crisis brings opportunity	Guidance
	Change practices	
Concepts (lived experience)	Networks & frameworks (community agency)	
Concepts (system)		
Researcher identity		
	Networks & frameworks (researchers)	
Human resource		Networks & collaborators

The themes are now presented in more detail, outlining the link from challenge, to solution, to support needs, and evidencing each theme through quotes from across the interviews. Quotes are labelled as follows: UK = UK-based participant, OS = overseas-based participant; M = male, F = female; world region; GCRF strategic portfolio theme. The symbol […] is used when, for economy of exposition, a portion of text is omitted mid-quote.

### Challenge 1: Low priority

Interviewees identified mental health impact as a low priority within their research area, as research output, by researchers themselves, and, where health is concerned “*the physical has been dominant*. *It’s easy to see a broken bone*” (UK, F, Southeast Asia, Health). It was also the impression that mental health is often a low priority in LMIC and a challenge to get onto the agenda with regard to related phenomena. For example, in relation to displace populations, one participant explained that it is an “*important thing to let them have mental health and this cultural side of life than just a sweater or something to eat*” (translator for OS, F, Americas, Education). Suggested solutions are ‘leadership and stipulation’ and to ‘link to a “sellable” priority and/or normal practices.’

#### Solution 1: Leadership and stipulation

Interviewees suggested that all the ‘big players’ needed to provide leadership in mainstreaming mental health: “*it’s a big problem*, *this high prevalence of depression here*, *and now it has gotten worse with the pandemic […] but still there hasn’t been any signal from the government*, *or from other agencies that there will be some funding particularly allocated for research and development in that area*” (OS, M, Americas, Education). Good examples were offered with regard to the appointment of mental health champions at national level and the leadership of agencies, such as the UK Office for Standards in Education, Children’s Services and Skills in the use and valuing of wellbeing measures. Funders could also stipulate incorporation of mental-health-based activities and reporting rather than consider it subsumed under ‘ethics’ or ‘safeguarding’: “*I think it’s too late for gateways*. *We shouldn’t make this palatable*. *I think it’s like*, *you have to do this*” (UK, F, Africa, Health).

#### Solution 2: Link to a ‘sellable’ priority and//or normal practices

To embed mental health impact across SDGs, one solution is to find ways to link mental health to the central aims of diverse projects. Interviewees could see many links, for example, to the ubiquity of living with uncertainty, tenuous citizenship, and food (in)security: “*The most common way is let’s take the national data that’s collected from*, *you know*, *food input/output*, *all that*, *where you don’t talk to people*. *And let’s work out what’s going on and let’s say okay there’s enough food and there’s enough nutrients*, *so everyone should be fine*. *And then there’s the other side*, *which is the side I*, *you know*, *am on which is where I actually talk to people and say*, *okay*, *how you’re experiencing food security*, *what is going on in this household scale or the community level*, *and it’s in that sphere that you’re going to come across where people are really looking at this mental health aspect because that’s where they can capture it*” (OS, F, Western Pacific, Planetary Health). Many key foci across SDGs also have implicit mental health impacts such as education, support for girls and women, healthcare, and strengthening families: “*saying that I’m talking about mental health*, *they will say ‘What*? *Oh that’s about mental illness*. *We only have small number of people that suffering of this’*. *Something like that*. *But when we start talking about family wellbeing first they will say ‘Ooh yeah*, *this our problem*. *We do have so many people having divorce*, *family violence*’” (OS, F, Southeast Asia, Health). The impact of psychosocial wellbeing extended even to understanding herding communities’ use of weather forecasts: “*accepting that people*, *when they’re given forecasts*, *don’t often react completely*, *how to put it*, *I suppose*, *economically rationally*, *yes*, *that we have- so there’s other things going on that determine how people behave […] just trying to understand people’s rationality for why they would move and why they wouldn’t move so there’s a concept of being trapped in a particular location and trying to understand their subjectivities”* (UK, M, Africa, Planetary Health).

#### Support need: Advocacy

Many agreed with the perspective that we have “*to change the kind of paradigm of development […] we have to inform the kind of young people going into this field that mental health is part and parcel of every single thing that we do”* (UK, F, Africa, Health). However, the perception is that “*it’s a vicious cycle*. *We don’t have funding so we don’t have a robust resource*, *so that’s why we do not have*, *you know*, *the data of policy relevance and we also do not have an active advocate group within the mental health field”* (OS, M, Eastern Mediterranean/ Southeast Asia/Africa, Health).

### Challenge 2 & 3: Evidence & tools

Interviewees noted a deficit of evidence about mental health needs in LMIC, particularly a lack of baseline data, and were uncertain what tools to use. Specifically, in terms of evidence, there is perceived to be a lack of data in a data-driven system: “*they’re drinking methylated spirits afterwards and I was like ‘what*?*’ and I couldn’t believe it*. *So I’m talking around and I’m talking to people and this was nothing to do with my research*. *My research was food but I thought this isn’t good and I reached out to*, *you know*, *doctors and nurses and they were like*, *well it goes on a little bit*. *And then I*, *you know*, *got higher up the chain and I spoke*, *you know*, *the medical director of health for that region and I said what’s going on and he goes ‘Oh no*. *That doesn’t happen’”* (OS, F, Western Pacific, Planetary Health). Not only was it not clear *what* to measure but *how* to measure mental health impact, and there is a perceived lack of short, appropriately validated instruments: “*The most comprehensive tool I found was a World Bank tool and it was about a 50 page questionnaire and I just thought*, *we can’t really use a tool like that to encapsulate what we’re trying to measure here*” (UK, F, Western Pacific, Health). Flexible methods were suggested as a possible solution.

#### Solution: Flexible methods

In terms of quantitative measures, it was suggested that very basic indicators to capture psychosocial wellbeing are needed. However, interviewees were open to considering the value of qualitative data, visual mapping, and participatory community methods. For example, one interviewee described the use of participatory geographic information system mapping: “*we asked people to identify*, *kind of*, *important people for them and if that changed when there was a flood going on*. *So we were able to map these relationships and put value on them*, *or rather*, *describe why those were important to people and what happened through those relationships*. *And then we could put that onto a GIS of city drains and flood risk zones […] the city planners were really interested in that*” (UK, M, Africa/Western Pacific, Health). In order to incorporate greater flexibility in the research methods used, it was suggested that funders needed to consider innovative ways to support long-term impact: “*ethnographic research- so I’m with the people for quite a long time and really involved in their stories*, *and they- and their relationships carried out after the PhD*, *and after the research*” (OS, F, Americas, Education). And to allow within-project responsiveness: “*more scope for that flexibility*, *that is something that you are working*, *you know*, *understand the mission of the project*, *something that is work in progress*” (UK, F, Americas, Education). Interviewees suggested they needed support by way of ‘data and evidence’ and ‘research tools.’

#### Support need 1: Data and evidence

Interviewees suggested a stakeholder analysis and consultation is needed to determine the range of mental health impacts possible in LMIC and, conceptually, more clarity on what counts as evidence, anxious that what they have is too ‘anecdotal’: “*just from quotes from the mediators we interviewed*. *They are saying*, *you know*, *that people felt a lot better or their mood improved or they felt more hopeful*” (UK, F, Eastern Mediterranean/Americas, Health). Finally, the evidence-base needs to be brought together and to consist of strong, relevant data, systematically-collected: “*maybe a member of staff during collection of data*, *they noticed it*, *but if it’s not on their questionnaire*, *it wasn’t ticked*, *there wasn’t such a*, *you know*, *a question or a box to tick*, *the information will probably just be lost*, *you know*, *during*, *in the process*. *So there isn’t such a mechanism to pick it up*” (OS, F, Western Pacific, Health).

#### Support need 2: Research tools

The type of tools required is a contentious area. Some interviewees welcomed conceptual clarity to allow the development of relatively straightforward, culturally-appropriate and validated measures for assessing impact. Others perceived greater need to raise the status of qualitative tools, such as a formal set of questions to assess stress, but also guidance on more involved methods: “*a lot of people do talk about participatory research*. *All of the tools are not necessarily as clear and so then it became interesting because we were developing and designing those as we went along*” (OS, F, Europe, People & Societies). It was also deemed important to be able to assess the added value of embedding mental health impact in a project: “*co-benefits that allows people*, *children to go to school*, *people to earn a living*, *all those things*. *But how could you even evidence that because ‘A’ you never set out at the beginning to evidence at the end*. *‘B’ you’ve never done that before and measured long-term outcomes”* (UK, F, Global, People & Societies).

### Challenge 4: Timeframe

The limited timeframe of research projects was viewed as a challenge to incorporating mental health impact. Interventions can be needed immediately, e.g., with populations in the process of displacement, while impacts can take time to manifest: “*we’d begun to bring that community into our- into our trust and then we weren’t able to follow-up*” (OS, F, Southeast Asia, Health). Moreover, regional instability can make it difficult for initiatives to embed, retain policy support, and reach sustainability: “*she was interviewing political leaders and realised that although they all knew what the rules and the policies should say*, *what actually happened on the ground was a million miles away*, *you know*, *it just hadn’t really embedded yet*” (UK, F, Southeast Asia, Health). Suggested solutions are to leverage ‘crises to bring opportunities’ and to ‘change practices.’

#### Solution 1: Crisis brings opportunity (interventions need to be fast).

Almost paradoxically, the tight timeframe of crisis situations could provide opportunities to innovate mental health impact. Interviewees reflected on how this can be facilitated in contexts where there is particularly fluid governance structures and when disasters catalyse awareness about psychosocial wellbeing, including the strain on healthcare workers: “*the first mental health cluster that was established in the Philippines […] the cluster system which in the way came into function sort of around the time of the Indian Ocean tsunami around 2005 was a way to kind of be able to co-ordinate better between sectors and between organisation*” (UK, F, Global, People & Societies). A more recent example is the way that “*issues of mental health and wellbeing in general starting to come up more strongly throughout the pandemic*” (OS, M, Americas, Education).

#### Solution 2: Change practices (fast interventions versus long term impact)

A second solution to the limited timeframe of research projects is to change practices. For LMIC and the research projects in these settings, lack of resource includes, effectively, lack of time to engage conventional mental health interventions, in part, because they are not available in the locale. One way forward is to develop community-led solutions which are responsive to local challenges: “*an unusual study actually asking survivors and they came up with*, *this is a couple of years ago*, *they came up with four or five key issues which led to an agenda called Survivor Led Response Reconstruction which is getting quite influential around humanitarian practice now and one of those five was around psychological health and wellbeing*” (UK, M, Africa/Western Pacific, Health). This includes conceptualising mental health impact as a kind of ripple effect: “*we have 10*,*000 of primary healthcare clinics […] but we don’t have mental health services there*. *So we are starting from one district and currently maybe more than five district*, *six district*, *yeah*, *so very slowly accepting this idea because it is I mean like new spending on their budget*” (OS, F, Southeast Asia, Health).

### Challenge 5: Concepts (lived experience)

There was discussion of the ways in which wellbeing is experienced and conceptualised across the many cultures and contexts of LMIC. Professional concepts may be too abstract for those outside the mental health field and terminology for describing lived experiences can be context-dependent: “*there the definition of kind of poor mental health they used actually an English word ‘tension’ and you know they’d say ‘Oh I had tension about’ for anxiety”* (OS, F, Southeast Asia, Health). Interviewees saw a need to work with local understandings but also conveyed how this can take time to achieve: “*if you’re thinking sort of more psychosocial*, *as we would be*, *then it’s got to be embedded in the context and you’ve got to know the situation of the geography*, *the politics*, *the culture*, *and just inside that community*” (UK, F, Southeast Asia, Health). Understanding the local context also involves sensitivity to mental health stigma towards social groups and fear of stigmatisation. A potential solution may lie in building on community networks and thinking in terms of locally-contextualised frameworks.

### Challenge 6: Concepts (system)

Interviewees considered the multiple, and often competing, concepts associated with mental health across and within health systems and academic disciplines and how we do not have a conceptual model or theoretical framework that captures wellbeing at this broad level. Interviewees debated the complexities around concepts such as ‘resilience’ or ‘trauma’ indicating that it can mean different things in different disciplines: “*trauma means something different for a historian than a psychologist*” (UK, F, Americas, People & Societies). They also noted that a key distinction is the apparently dominant positioning of mental health within psychiatry, with perceived emphasis on pathology, or within public health, with perceived emphasis on wellbeing and enabling civic engagement: “*I think that there needs to be more cross-disciplinary conversation and especially if we’re thinking about certain professions*, *like welfare workers*, *you know*, *social workers*, *psychologists in schools*, *so there’s a recognition that there’s still so much work that needs to be done here*, *along those lines*, *but also in how some of us who have stayed away from those questions could ask then in ways that are beneficial to our work*” (OS, F, Europe, People & Societies). Additional contested positions identified are around mental health as a commodity, as a right, and/or as a condition of quality of life: “*They’re sort of questioning whether it shouldn’t be called Shelter and Settlements*, *it should be called Homes and Communities*. *So*, *there’s that*, *there is a sort of growing awareness of the fact that shelter doesn’t represent humanitarian shelter […] more about how do people recover their home and all that represents*” (UK, F, Global, People & Societies).

Again, solution looked to facilitating community agency with respect to the meso- and macro-context and instigating networks of professionals and local leaders to identify frameworks relevant to the local environment.

### Challenge 7: Researcher identity

We were unable to recruit an interviewee on an STFC GCRF grant or non-UK interviewees on GCRF grants related to GCRF strategic portfolios (i) Cities & Sustainable Infrastructure, and (ii) Resilience to Environmental Shocks & Change. This difficulty may reflect what some interviewees said with regard to the probability that embedding mental health impact in their work would be an alien proposition to many researchers in these fields. One issue is that mental health may be perceived as a professionalised area: “*the dancing and all those group activities*, *I don’t know enough about mental health and young people and traumatised young people to know how that relates to what they would normally do or how things are supposed to be*” (UK, F, Africa/Europe/Global, People & Societies). Interviewees also raised ethical concerns about the possibility of doing harm and/or of doing something too superficial: “*they are hit and run*, *you know*, *however you do with it because your project funding is only for a few years*, *unless you’re involved in one of those long-term observatories*, *you come*, *you collect your data*, *you write your paper and however good a person you are*, *you don’t have a long-term positive impact*” (UK, M, Africa, Planetary Health). On the other hand, they also reflected on practices in some disciplines which may provide some resonance with the idea of making a mental health impact: “*the psychologist’s subjective positions when you are doing your research*. *And I wonder if it is quite common in social science subjects*, *but it’s probably not*, *not as common in*, *in you know*, *non-social science*” (OS, F, Western Pacific, Health). Again, networks and frameworks were discussed as possible solutions, both in terms of working with local communities and as a community of researchers.

#### Solution 1: Networks and frameworks (community agency)

Networks and frameworks relevant to local communities were viewed as possible solutions with regard to challenges 5, 6 and 7: that is, concepts (lived experience), concepts (system), and researcher identity. This involves the local community taking the lead in identifying mental health needs: “*what we wanted was to go back and work with the people that we started with and to really understand what it was they wanted rather than what we wanted to give them*” (UK, F, Southeast Asia, Health). Moreover, creating innovative solutions through leveraging existing local channels of communication and support: “*these communities normally have very elaborate traditional coping mechanisms*. *The most key of them is then social capital*, *that they have very strong social alliances between the community and also across the community”* (OS, M, Africa, Planetary Health). To facilitate such initiatives, researchers need appropriate theoretical frameworks such as person-centred principles and an ethics of care approach, and practical frameworks such as participatory methodologies to create a bridge between local and institutional knowledge. In this way local people such as teachers, religious leaders, health professionals, parents, and older school children might be trained to identify people in distress and to signpost, or even provide basic, support: “*just identifying it won’t be a solution*, *I mean there should be a proper channel*, *at least if we are not able to do it into the greater extent*, *maybe in the lesser extent*, *or just talking and sharing*, *and some kind of session would be like really nice*, *I think yeah*. *I think in most of the mental health issues I think you do not need a very sophisticated mental health support”* (OS, F, Southeast Asia, Health).

#### Support needs: Guidance

A key support need was for guidance with the observation that, if embedding mental health impact takes off, “*a whole industry will rise up about*, *you know*, *supporting this*” (UK, F, Africa, Health). Guidance was required in relation to challenges associated with ‘timeframe,’ ‘concepts,’, and ‘researcher identity.’ Suggestions included the provision of evidence through scoping reviews on mental health in LMIC and retreats for researcher debrief and skills dissemination. Training requirements included that on: mental health impact (e.g., in preparing grant applications, different ways of taking a wellbeing perspective in your research setting, working with local partners); interventions (e.g., how to operationalize emergency interventions, and how to adapt interventions that can cross contexts); local capacity building (e.g., guidance for developing community support groups); and how to identify local perceptions and practices of wellbeing: “*I think an awareness of how trauma works […] So in terms of training*, *I think that would help*. *I think because of the type of work that I do*, *I would say more than training*. *It’s important to have the possibility to engage in long-term relationships with the participants*” (OS, F Americas, Education). Although a good starting point, some were of the opinion that those who might engage with the provided guidance are probably those already attuned to mental health.

### Challenge 8: Human resource

A very practical challenge of incorporating mental health impact across diverse projects is the lack of personnel to undertake this work: “*GCRF ought to underwrite this more because*, *actually*, *we’re not paid to do it*” (UK, F, Southeast Asia, Health). Some reflected on the perception that wellbeing impact may by default appear to fall within the remit of the qualitative researcher but that there needs to be capacity and skills across the team to look at data through the lens of mental health. This may avoid the risk of researcher overload and for mental health impact to become a ‘tick box’ exercise. Another issue raised is that, in some LMIC contexts, there may be no local psychologist or psychiatrist and no-one suitable for wellbeing training: “*there are some hospitals who provide the helpline*, *if you have any like suicidal thoughts or any major psychological and mental health issue*, *but these are also limited*” (OS, F, Southeast Asia, Health); “*not many organisations actually do mental health*, *whatever that means*” (UK, F, Global, People & Societies). A possible solution is to build networks of researchers and provide frameworks with which to work.

#### Solution: Networks and frameworks (researchers)

Networks and frameworks for researchers was viewed as a possible solution with regard to challenges 7 and 8: that is, researcher identity and human resource. Interviewees suggested that what is needed is a community of researchers who value wellbeing. Sometimes the need for researcher networks had been identified in-house: “*within our institute*, *those of us who are interested are going to try and develop some very basic listening and counselling type of skills and then not to be properly trained counsellors for deep you know*, *psychological problems but just to be able to*, *if someone’s dealing with sensitive data that is upsetting that they would you know*, *have an established sort of buddy or a peer listener who could talk through with them*” (OS, F, Southeast Asia, Health). More generally, the situation was that “*we really realised that mental health and support*, *peer support or colleague support etc*. *is a real gap in the sector”* (UK, F, Global, People & Societies). However, a shared language framework is also needed to help researchers identify with embedding wellbeing impact in their work, including their own wellbeing.

#### Support needs: Networks and collaborators

To help address the human resource challenge, under the assumption of little or no additional resource, we might look to researcher support networks and to collaborators.

It is important to have the right people in place, the partners on the ground, and to be able to refer to a local provider, if possible: “*we had all these connections through [partner organisation] and this international relation and so we could sort of get started relatively quickly […] you’d need a pretty long-term perspective wouldn’t you to do something sensible on*, *I don’t know*, *on mental wellbeing aspects*” (UK, M, Southeast Asia/Western Pacific, Planetary Health). In particular, in-country networking and collaboration with mental health specialists can smooth the way. Researcher networks may be able to provide mentoring, training opportunities within the network, and collaboration with groups for whom mental health is the main focus: “*a local kind of peer level support system where you know I can say*, *a colleague can write to me knowing that I’m on the list of peer responders and say you know*, *‘I could really do with a chat’*, *and I can go*,*‘Yes I’m here*, *anytime*, *here’s my time slots’*” (OS, F, Southeast Asia, Health).

## Theory of change

Based on the conceptualisations developed in our thematic analysis, we created a ToC designed to promote the mainstreaming of mental health in global challenges research. To do so, we followed the five steps to defining a ToC as described by van der Laan et al [24]:

- Define your desired *impact*. This change at the level of end-users, communities or constituents understanding that, while these are in our sphere of interest, we can only contribute to it.
- Determine your *outcomes*. This refers to change in behaviour, relationships, actions, activities, policies or practices of an individual, group, community, organisation, or institution. Outcomes describe which specific local stakeholder is doing what differently within our sphere of influence.
- Identify your *pathways* of change.
- Specify your *strategies*. This is a general description of what the programme needs to do to make the expected *outcomes* happen.
- Connect your *pathways* of change.

**Fig 1:**
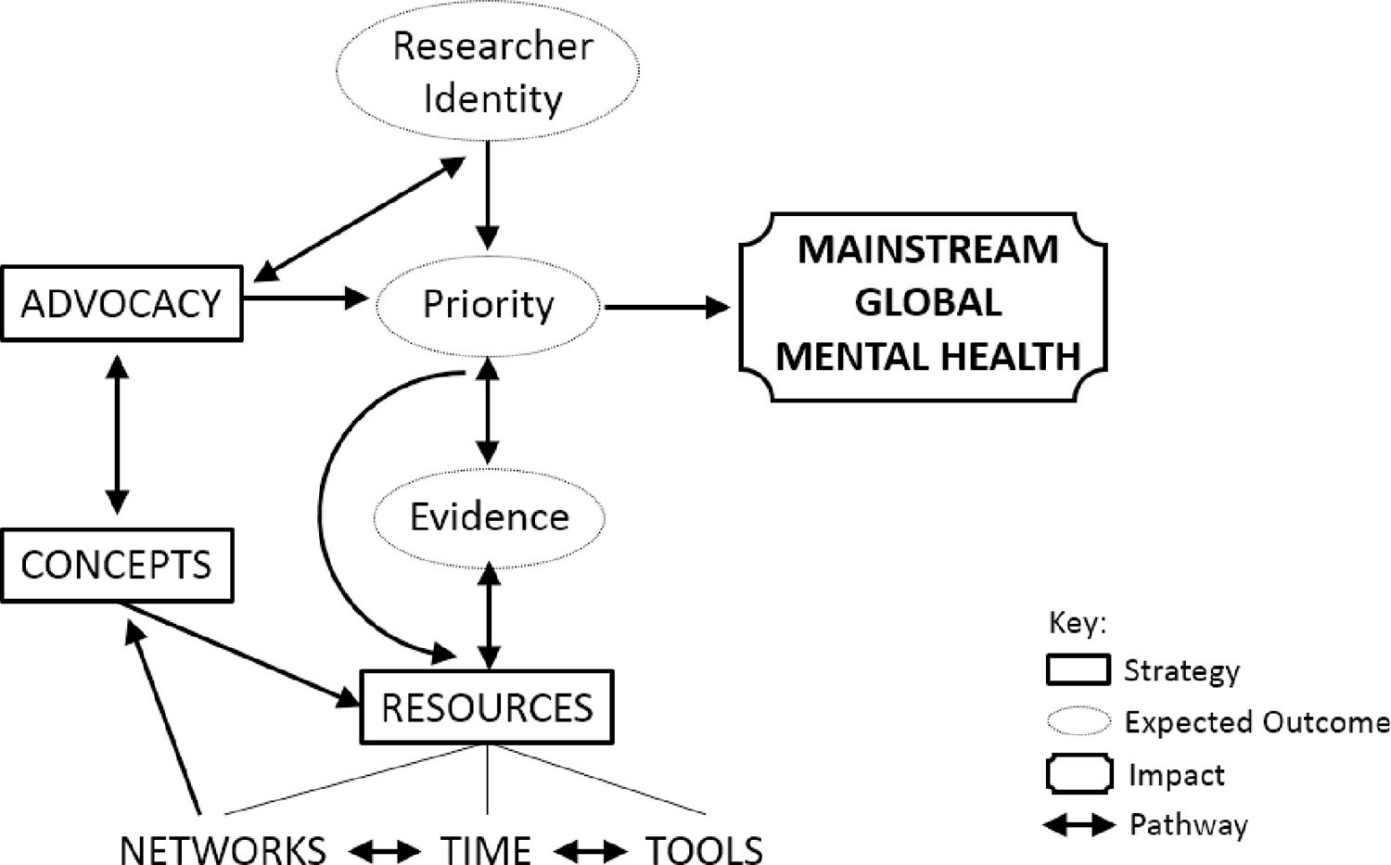
Theory of change to promote mainstreaming mental health in global challenges research.

The first author defined the intended *impact*, identified themes from the analysis which could be considered *outcomes* and *strategies*, and posited the *pathways* between them. She then presented the resulting ToC in diagrammatic form to the authorship team. The theory was interrogated in detail leading to the revelation of gaps and the need for some revisions. For example, the original theory did not incorporate all ‘*needs’* identified in the interview analysis and did not have a feedback loop allowing for in-built sustainable, agile adaptation. The first author revised the theory accordingly and it was accepted as credible and realistic by the authorship team with some additional minor changes. The ToC is provided in Fig 1 and described below. The links to the thematic analysis are highlighted throughout identifying where the elements of the ToC had been identified as a ‘*challenge’*, ‘*solution’*, or ‘*need’* in the original thematic analysis.

### Impact

At the level of end-users and communities, the change sought is to *mainstream global mental health*. This is in our sphere of interest and we can contribute to making this change happen.

### Expected outcomes

To effect a sector culture change towards an increase in the mainstreaming of global mental health, the following changes need to occur and these expected outcomes are in our sphere of influence.

#### Priority (‘challenge’)

An expected outcome is to *increase the priority given to mental health*. This will require a change to the policies of relevant organisations, and a change in the practices of individual researchers and research groups, endorsed by the research community (‘*solution’*). What specific local stakeholders will do differently is, through leadership and stipulation *(‘solution’)*, develop policies at organisational level which facilitate the mainstreaming of global mental health impact which are enacted by individual researchers and research groups. This is the direct *pathway* to *impact* through which there will be an increase in the mainstreaming of global mental health.

#### Researcher/Agent-of-change identity (‘challenge’)

Our study focuses on GCRF researchers and their collaborators as the relevant agents-of-change. However, we anticipate potential agents-of-change to include researchers and development workers in any country, on any funding stream, and within any organisation who are working to deliver the SDGs. An expected outcome is to *increase the number of researchers who identify mental health impact to be within their remit*. This will require a change in the behaviour, relationships, actions, activities, and practices of individual researchers, research groups, and the research community, facilitated by relevant organisations. What specific local stakeholders will do differently, i.e., the researchers designing and implementing global challenges research projects, is to embed mental health impact in their work from the start, understanding how this contributes to delivering their primary aims. They will do this, for example, through linking mental health impact to a ‘sellable’ priority (e.g., food security) and/or normal practices (e.g., fieldwork interactions) (‘*solution’*). These researchers will increase the priority given to mental health impact in their work and are a *pathway* through which the case is made to increase the priority given to mental health impact at an organisation, state, and global level.

#### Evidence (‘challenge’ & ‘need’)

The final expected outcome is to *increase the available evidence with regard to global mental health impact*. A bi-directional *pathway* exists between evidence and priority such that greater prioritisation of mental health will increase the need for evidence, and increased evidence will support the need to give greater priority to mental health. A second bi-directional *pathway* exists between evidence and resources such that the need for evidence will increase the need for resources supporting mental health impact, and the increased availability of relevant resources will facilitate the development of evidence.

### Strategies

For the *expected outcomes* to happen, action needs to be taken with respect to resources, concepts, and advocacy.

#### Resources

Appropriate resources supporting mental health impact need to be available and accessible. Resources consists of *tools (‘challenge’ & ‘need’)*, *time (‘challenge’)*, and *networks* (‘*solution’* & *‘need’)*. *Tools* include all forms of guidance *(‘need’)*, including frameworks (‘*solution’*), quantitative and qualitative approaches, and other flexible methods (‘*solution’*) of data collection *(‘need’)* and analysis to impact global mental health. *Time* includes the pressured trajectory of research projects, LMIC regional and governmental instability, the need for immediate psychosocial interventions, and the necessity for long-term sustainable activities. However, time pressures during a crisis can be an opportunity for positive innovation (‘*solution’*). *Networks* are needed to offset the limited available human resources (*‘challenge’*), to find collaborators *(‘need’)*, to share skills and knowledge, and to provide mentorship within the research community and promote the agency of local LMIC communities (‘*solution’*). A bi-directional *pathway* exists between *tools* and *time* and between *networks* and *time* such that appropriate *tools* and effective *networks* can offset the lack of *time*, while more *time* will allow the use of more and/or different *tools* and greater engagement with *networks*. As well as a bi-directional *pathway* between evidence and resources, as outlined above, a uni-directional *pathway* exists such that greater prioritisation of mental health will increase the need for resources.

#### Concepts (‘challenge’)

Relevant concepts, and ways of working with multiple locally-appropriate concepts, need to be developed with regard to global mental health. This is required in terms of lived experience in different LMIC contexts and in terms of the multiple and competing concepts across and within health systems and academic disciplines. Developing relevant concepts is a *pathway* informing the development of appropriate *resources*. On the other hand, a *pathway* for developing relevant concepts exists from the *resource* of community and researcher *networks*.

#### Advocacy (‘need’)

Advocacy in the form of championing the mainstreaming of global mental health is required. A bi-directional *pathway* exists between advocacy and concepts such that greater clarity and ways of working with multiple locally-appropriate concepts will inform an effective advocacy message. Additionally, knowledge exchange during the advocacy process will improve conceptual clarity. In particular, knowledge exchange achieved in the advocacy process with researchers/agents-of-change is a bi-directional *pathway* encouraging identification with the remit of effecting mental health impact while providing feedback on conceptual clarity. Finally, advocacy is the main *pathway* to increase the priority given to mental health, facilitated by the *pathway*, described above, of increased numbers of researchers/agents-of-change identifying with this impact.

## Discussion

In this article we ask: How can we mainstream mental health in research engaging the range of SDGs? Our thematic analysis of 26 interviews with GCRF researchers and their collaborators identifies a trajectory from the challenges of incorporating mental health impact into the kind of work they do, to how these challenges might be overcome and, finally, to support needs. This analysis was then organised into a ToC designed to promote sector culture change towards the mainstreaming of mental health in global challenges research. These findings are now considered in relation to the relevant literature, reflections on the work, and implications for researchers, mental health practitioners, and global challenges research funders.

### Challenges, solutions, needs

Regarding the challenges identified, it is recognised increasingly that the mental health impact of humanitarian crises are often unattended [25], that effective strategies need to orient to the long-term [7], and that there is a lack of data on global mental health or, as is our contention with regard to global challenges research across the SDGs, evidence of the impact which is already being made through routine project activities [15]. As Ryan et al state: “If indicators on psychosocial disability, mental health, and wellbeing were regularly used, many more development activities would likely come to be recognised as effective psychosocial interventions” [6 p17]. However, the Department for International Development (DFID) [7] acknowledges the reticence of some development practitioners to engage mental health impact as outside their remit, and/or skill, in their call for tools to minimise the risk of doing harm. Finally, there is an ongoing search for ways in which to conceptualise ‘mental health’ as lived experience, a field of professional practice and, increasingly, a state responsibility. Tensions exist amongst these knowledges, particularly on the global scale, with theoretical debates spanning the bio-socio-cultural spectrum and potentially beyond [26].

In terms of the solutions identified, leadership is becoming evident through initiatives such as the *Convention on the Rights of Persons with Disabilities* [27], the signatories of which “are obligated to ensure that their overseas development and humanitarian programmes are inclusive of and accessible to people with disabilities” [6 p14]. In fact, human rights appears to be the dominant framework currently for promoting global mental health impact. In the UK, the Department for International Development and the Foreign, Commonwealth and Development Office *An Approach and Theory of Change to Mental Health and Psychosocial Support for Global Development Actors* [8] provides a strategy highlighting, as did many of our interviewees, that emergency situations can present an opportunity to strengthen mental health systems [28]. In fact, WHO issued a newsroom factsheet on 16^th^ March 2022 stating that “despite their tragic nature and adverse effects on mental health, emergencies have shown to be opportunities to build sustainable mental health systems for all people in need” [29, para 5] and detailing positive case studies in the Syrian Arab Republic, Sri Lanka, Philippines, and Caribbean.

The solution of building on community networks is commensurate with DFID’s guiding principle for development practitioners to put “people at the centre of decision making on their own health and lives—as well in policy, research, and services” [7 p6] and the importance of collaboration within, and between, sectors and civil society [30]. Finally, of extreme relevance to development researchers, Ryan et al [6] provide an evidence summary of how mental, neurological, and substance use conditions can be framed as part of the SDGs. This supports the solutions we identified of linking mental health impact to a ‘sellable’ priority and/or ‘normal practices.’ More work is needed to understand how to mainstream mental health across global challenges research in terms of ‘changing practices’ and the acceptability and use of more ‘flexible methods’ identified as potential solutions in our analysis.

In terms of needs, DFID [7] note the lack of data and evidence on global mental health, that more research tools and guidance are needed, and that advocacy is required to champion user co-development and coordination with services and sectors beyond healthcare. Moreover, as indicated in our study, DFID recognise a need to “(i)nclude the well-being of humanitarian staff and volunteers in organisational development plans” [7 p17]. A start is being made by Ryan et al’s [6] *Mental Health for Sustainable Development*: *A Topic Guide for Development Professionals* which includes guidance on stepped care, the intervention pyramid, and evidence on mental health interventions which can be provided by trained and supervised community workers [31].

In summary, many of the challenges, solutions, and needs identified in our interviews with GCRF researchers and their collaborators are noted in recent and emerging global and national policies and initiatives designed to raise the priority of mental health as a development goal. Importantly, mental health impact is increasingly viewed as an appropriate aim beyond the remit of the mental health specialist and a start has been made in providing guidance and resources to support this vision in the development and humanitarian sectors. Global challenges funders and researchers are a distinct set of stakeholders who have not yet been invited directly to consider mainstreaming mental health.

### Theory of change

**Fig 2:**
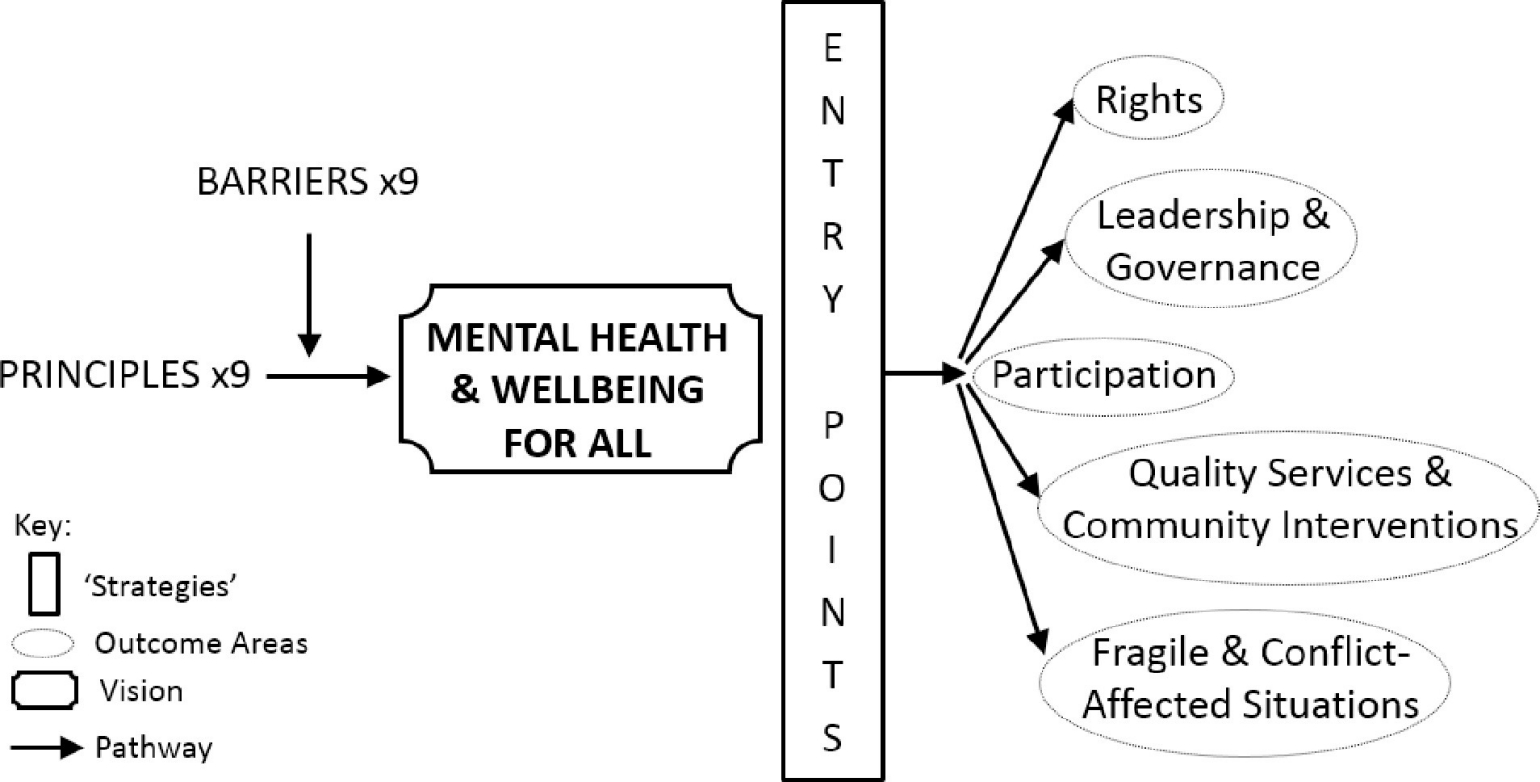
Theory of change to support global development actors deliver mental health support (from DFID, 2020).

If we wish to mainstream mental health in research engaging the range of SDGs, we need a map to guide us towards this goal. Hence, we posited a ToC based on the conceptualisations in our thematic analysis (Fig 1). The most relevant existing ToC is that of DFID [7] designed to support global development actors deliver mental health and psychosocial support (Fig 2 created by the current authors). Their vision is that “All people enjoy the highest attainable standard of mental health and wellbeing and all people with mental health conditions and psychosocial disabilities can exercise their full rights on an equal basis to others” [7 p5]. The desired change of our ToC–our vision–is the mainstreaming of global mental health within the global challenge research sector. These visions are commensurate, the present article identifying, and extending into, additional stakeholders.

The two ToC are structured using similar concepts, although labels for the various elements vary: impact/vision, outcomes/outcome areas, pathways, and strategies/entry points for change. In addition, the DFID ToC specifies ‘principles’ and ‘barriers’. However, in both, the process of change is conceptualised as multidirectional and context specific, and both are situated with reference to the UN SDGs and the WHO mental health action plan. DFID draws also on the CRPD and this is reflected in the way their vision of mental health and wellbeing for all is grounded in nine guiding principles: participation; human rights and dignity; comprehensive, integrated and responsive services; quality; collaboration and partnerships within and across sectors; equity; do no harm; context is critical; and, data, monitoring, evaluating and learning. While the concept of ‘barriers’ is not explicit in our ToC, it is implicit in that ‘challenges’ identified in our thematic analysis appear in its various elements. Using the language of the DIFD ToC, the most direct overlaps between their ‘barriers’ and our ‘challenges’ are: lack of skilled workforce; lack of data and information; and, lack of sustainable resources and political will.

The DFID ToC specifies five ‘outcome areas,’ each constituting a ‘critical pathway for change’ with identified ‘entry points.’ The first outcome area is deemed central to all the pathways: “full and meaningful participation of people with mental health conditions and psychological disabilities in decision-making” [7 p7]. The other four outcome areas are: rights; leadership and governance; quality services and community interventions; and emergencies and fragile and conflict-affected situations (FCAS). The ToC in the present article identifies three outcomes with regard to global challenges research, i.e., our area of focus: to increase, (i) the priority given to mental health; (ii) the number of researchers who identify mental health impact to be within their remit; and, (iii) the available evidence with regard to global mental health. In line with our more tightly proscribed stakeholder groups, our expected outcomes are similarly focused while compatible with those of the DFID ToC.

For the *expected outcomes* to happen, action needs to be taken with respect to resources, concepts, and advocacy. We posit three strategies, i.e., actions that need to be taken, to fulfil our outcomes: appropriate resources supporting mental health impact need to be available and accessible; relevant concepts, and ways of working with multiple locally-appropriate concepts, need to be developed; and, advocacy, in the form of championing the mainstreaming of global mental health, is required. The DFID ToC identifies multiple, critical entry points for change relevant to each outcome area. An illustrative example with reference to the foundational outcome ‘participation’ is as follows: that “people with mental health conditions and psychosocial disabilities are empowered to advocate and meaningfully participate in decision-making and in the design and implementation and monitoring of legislation, policies, strategies and services” [7 p9]. Three entry points are identified as critical to ensuring participation across all five pathways of change: establishing representative organisations; enabling meaningful inclusion and decision-making; and, user-involvement in system strengthening.

The participation of people with mental health conditions is emphasised by DFID. It is implicit in our feedback loop between *Resources* (networks) and *Concepts* and encourages dialogue around diverse framing of ‘distress,’ but we could consider how to forefront better the principle of inclusivity in the application of our ToC. On the other hand, our ToC does have scalability, at least in principle, and can be implemented by the individual researcher and within individual projects but also by organisations, international sectors, and incorporated into government policy initiatives. This is a huge strength and means that its pathways can be adapted for the spheres of influence of those driving change.

In summary, the ToC posited in this article is commensurate with that presented by DFID [7] which seeks to extend the remit for mental health impact across the development sector. Our ToC is focused on a more proscribed area, i.e., research engaging the range of SDGs, and related stakeholder groups such as organisations funding, and researchers undertaking, global challenges projects. These are stakeholders addressed only indirectly by the DFID initiative and, as suggested by our work, associated challenges, solutions, needs, and pathways to change are not identical to that of development professionals.

### Reflections and implications

The present research is funded by the GCRF and the pattern of projects funded by this programme constitutes a major focus of this article. The impetus for this study is our own, the intention to conduct this work was articulated in our funding application, the work was conducted independently of the GCRF, and the fact that we are funded by the GCRF has not knowing affected our analysis or conclusions. Our team of 14 researchers brought a productive diversity of experience and outlook to this work in terms of career stage, academic discipline, and demographic characteristics, with representation from seven countries of origin–nine if we include our three mentees. Thirteen have prior project funding from the GCRF and, although this could have biased us in terms of our personal experiences, it also brings a wealth of knowledge about how the programme works and associated challenges.

Despite efforts, we were unable to obtain an interview with a STFC PI or collaborator. The STRC focuses on “astronomy, particle physics, space science and nuclear physics and research in any other field which makes use of scientific facilities where access is provided, arranged or otherwise made available by STFC” [para 4: https://stfc.ukri.org/about-us/our-purpose-and-priorities/mission/]. Although by the end of May 2020 STFC had awarded 40 GCRF grants, the stretch between these grants and mental health impact may have appeared too large to enthuse researchers to take part in our study. We were also unable to secure a non-UK interview with a researcher working in two of the GCRF strategic portfolios: (i) Cities & Sustainable Infrastructure, and (ii) Resilience to Environmental Shocks & Change. However, the six GCRF strategic portfolios can be clustered into four themes at a slightly higher level of conceptualisation all of which are represented in our sample.

Global challenges funders and researchers are a distinct set of stakeholders who have not yet been invited directly to consider the implications of mainstreaming mental health. Implications for researchers include: recognising the potential of their projects to have psychosocial wellbeing impact in LMIC; encouraging them to take ownership of this aspect of their work by building mental health impact into projects from the start; and collaborating with local communities, researchers, and service providers to effect change. Implications for mental health practitioners include: developing a more inclusive and flexible language around mental health that bridges cultures and disciplines; providing tools and guidance that democratises the process of effecting psychosocial wellbeing impact in context-appropriate ways; and engaging with researcher networks to offer mentoring. Implications for funders include: engaging with the desire of researchers to contribute more broadly to the wellbeing of the communities with whom they work; supporting this through considering greater flexibility for within-project adjustment; innovating ways of supporting long-term impact; grappling with the on-the-ground challenges of real partnership working in LMIC; and providing leadership to drive the culture change required to mainstream mental health impact in research across the SDGs throughout the sector.

In 2021, the GCRF programme was hit hard by reduction in the UK Government Overseas Development Agency (ODA) budget. This had a devastating effect on many projects and the already vulnerable communities served. Fortunately, in the Autumn Budget and Spending Review 2021, it was announced that the ODA budget would return to 0.7% of Gross National Income and that spend on research and development will increase [32]. The promised resurrection of budget provides an opportunity to consider the benefits of mainstreaming mental health in the GCRF programme, and beyond, and to put into practice the Theory of Change we present here.
